# The Impact of LPS on Inflammatory Responses in Alpha-Tocopherol Deficient Mice

**DOI:** 10.1016/j.cdnut.2024.104416

**Published:** 2024-07-14

**Authors:** Megumi H Seese, Andrew J Steelman, John W Erdman

**Affiliations:** 1 Division of Nutritional Sciences, University of Illinois at Urbana-Champaign, Urbana, IL, United States; 2 USDA-ARS Children's Nutrition Research Center, Houston, TX, United States; 3 Department of Animal Sciences, University of Illinois at Urbana-Champaign, Urbana, IL, United States; 4 Department of Food Science and Human Nutrition, University of Illinois at Urbana-Champaign, Urbana, IL, United States

**Keywords:** vitamin E, *RRR*-α-tocopherol, *Ttpa*-null mouse, lipopolysaccharide, oxidative stress, inflammation, sickness behaviors

## Abstract

**Background:**

To facilitate the evaluation of vitamin E (α-tocopherol, αT) status on health outcomes, the αT transfer protein knockout (*Ttpa*^*–/–*^) mouse model has proved to be an effective tool for lowering αT body stores. Our previous study showed a further reduction in grip strength in LPS-treated *Ttpa*^*–/–*^ compared with wild-type (WT) mice during a 9-wk αT-deficient diet feeding period but did not find a difference in LPS-induced inflammatory response markers. Further optimization of this mouse model is warranted to determine the appropriate depletion period and biomarkers endpoints.

**Objectives:**

The objective was to examine whether 12 wk of an αT-deficient diet altered the inflammatory response 4 and/or 24 h after LPS injection in WT and *Ttpa*^*–/–*^ mice.

**Methods:**

WT and *Ttpa*^*–/–*^ weanling littermates were fed an αT-deficient diet *ad libitum* for 12 wk. Mice were then injected with LPS (10 μg/mouse) or saline (control) intraperitoneally and killed 4 (Study 1) or 24 h (Study 2) later. Concentrations of αT in tissues were measured via HPLC. Grip strength and burrowing were evaluated to assess sickness behaviors before/after LPS injection. Expression of genes related to inflammatory responses was examined via RT-PCR.

**Results:**

αT concentrations in the brain, liver, and serum of *Ttpa*^*–/–*^ mice were notably lower or undetectable compared with WT mice in both studies. Hepatic αT concentrations were further decreased 24 h after LPS injection. Grip strength was reduced at 4 h post-injection but partially recovered to baseline values 24 h after LPS injection. The expression of genes related to inflammatory responses were altered by LPS. However, neither measure of sickness behavior nor gene expression markers differed between genotypes.

**Conclusions:**

A 4-h LPS challenge reduced grip strength and resulted in an inflammatory response. At 24 h post-dosing, there was a partial, transitory recovery response in both *Ttpa*^*–/–*^ and WT mice.

## Introduction

Oxidative stress is associated with many abnormalities, such as lipid peroxidation, DNA and protein damage, inflammation, and neurodegeneration. Vitamin E (α-tocopherol, αT) is an antioxidant that helps to protect against imbalance caused by excess production of reactive oxygen species (ROS) and prevents lipid peroxidation and oxidative stress in cell membranes [1,2]. Conversely, αT deficiency results in elevated oxidative stress levels, an abnormal neurological phenotype, and an altered immune system [[3], [4], [5], [6]]. Thus, low αT status increases the risk of oxidative stress and inflammatory responses.

Vitamin E includes 8 molecules, which further comprises 8 stereoisomers (an *R* or *S* orientation at positions 2, 4′, and 8′). *RRR* αT is the most active form among vitamin E analogs because hepatic α-tocopherol transfer protein (α-TTP) preferentially transfers *RRR* αT via lipoproteins into peripheral tissues. Importantly, α-TTP is expressed in the brain, suggesting that this protein may play a specific protective role in vitamin E homeostasis and function in this organ [5,7,8]. Mutation of α-TTP in humans results in ataxia with vitamin E deficiency. Although vitamin E metabolism is affected by several proteins, such as the scavenger receptor, class B type 1 [9], and ATP-binding cassette A and G [10,11], α-TTP is the protein that specifically recognizes and transports αT [12].

To study the impact of vitamin E status on health and disease prevention, α-TTP knockout (*Ttpa*^*–/–*^) mouse model has proven to be an effective tool because of lower αT body stores in *Ttpa*^*–/–*^ mice compared with wild-type (WT) mice [[13], [14], [15]]. Most prior *Ttpa*^*–/–*^ mouse studies focus on the effects of αT status during aging, as this transgenic mouse model does not exhibit severe abnormalities or morphological outcomes until adulthood [[4], [5], [6]]. However, alterations in gene expression have been reported in younger mice [16]. Our laboratory has previously contributed to establishing the timeline of the neurological phenotype in *Ttpa*^*–/–*^ mice [17], yet there is not a determined optimal time point for studying relatively young adult mice. The current study employed this transgenic mouse model to assess the impact of αT deficiency on inflammatory and oxidative stress responses in the murine brain. Our previous studies [[14], [15]] showed that a single dose of intraperitoneal LPS injection resulted in decreased grip strength, lower immune cell levels in blood, and increased markers of inflammation in blood and tissues. Furthermore, we showed exacerbated grip strength deficit in 12-wk-old *Ttpa*^*–/–*^ compared with WT mice during a 9-wk αT-deficient diet study period [15] but did not find other genotype effects in any markers of inflammatory and oxidative stress response tested. Some research has shown that proinflammatory cytokine-induced sickness behaviors following an LPS challenge result in physiological and behavioral abnormalities, such as decreased general activities (for example, burrowing and grooming) and loss of appetite [[18], [19], [20]]. Chung et al. [21] have shown that αT supplementation reduced LPS-induced oxidative stress and inflammatory markers, such as hepatic malondialdehyde (MDA) and TNF-α levels, in male leptin-deficient obese mice, confirming the attenuated inflammatory response 6 h after intraperitoneal LPS injection (250 μg/kg, single dose). Studies from other laboratories used different LPS doses and post-dose timing of testing to examine the impact of LPS treatment on a variety of functional, biomolecular, and morphological outcomes [[22], [23], [24], [25]].

The combination of vitamin E deficiency using *Ttpa*^*–/–*^ mice with LPS administration alters inflammatory and oxidative stress responses. We previously compared the LPS dose difference (1 compared with 10 μg/mouse) and found similar inflammatory responses in both WT and *Ttpa*^*–/–*^ mice [14]. Schock et al. [26] also demonstrated LPS-induced oxidative stress and inflammation in plasma, liver, and lung of *Ttpa*^*–/–*^ mice 12 h post-injection (LPS intraperitoneal 10 mg/kg), although their results did not show significant impacts of lower vitamin E status on the inflammatory–immune response in *Ttpa*^*–/–*^ mice, compared with WT mice. We concluded that the absence of genotype effects in former studies was attributed to the relatively young age of mice, the feeding period of αT depletion, and/or the specific time chosen for evaluating endpoint measures.

Therefore, the goal of the first study was to examine whether a longer, 12-wk feeding period of an αT-deficient diet, followed by exposure to LPS, would alter the inflammatory response 4 h after LPS injection in *Ttpa*^*–/–*^ compared with WT mice (Study 1: the 4-h post-injection study). Second, to test the impact of a longer recovery time after LPS administration, we measured inflammatory responses at 24 h post-LPS injection (Study 2: the 24-h post-injection study). These times of post-LPS injection selection were based on our previous studies [14,15] and literature [[22], [23],^25^] to measure peak- or post-peak inflammatory responses. We hypothesized that longer αT depletion would result in enhanced inflammatory and oxidative stress responses and concomitant sickness behaviors in *Ttpa*^*–/–*^ as compared with WT mice. A combination of biochemical and functional outcomes was comprehensively examined to evaluate the impact of 12 wk of an αT-deficient diet in 15-wk-old WT and *Ttpa*^*–/–*^ mice. The findings of the present study advance our understanding of the development timeline of αT deficiency indicators/markers.

## Methods

### Mouse study design

The study received the approval for all animal procedures from the University of Illinois Institutional Animal Care and Use Committee (IACUC #20180). Male *Ttpa*^*–/–*^ and control WT littermates were generated using a trio-breeder strategy (1 male *Ttpa*^*–/–*^ mouse and 2 female C57BL/6J mice/cage) per previously described procedures [14]. This study used exclusively male mice to remove sex as a possible variable because female and male mice had previously been shown to have differential αT accumulation in various brain regions [27]. C57BL/6J mice (RRID:IMSR JAX:000664.) were purchased from the Jackson Laboratory. *Ttpa* heterozygous breeders were fed an AIN-93G-based, low αT (LOW) diet [35 mg *RRR*-α-tocopherol acetate (αTA)/kg diet] to minimize brain αT accumulation in the offspring. The composition of experimental diets can be found in Supplemental Table 1. The offspring genotypes were confirmed via PCR with specific primers for *Ttpa* as previously described with slight modifications [28]. At weaning (3 wk of age), study mice were individually housed in shoebox cages (12:12-h light-dark cycle, 22°C, 60% humidity) and provided an αT-deficient diet *ad libitum* until study termination at 15 wk of age (Supplemental Figure 1). Weekly body weight and food consumption were measured throughout the experimental period.

**FIGURE 1:**
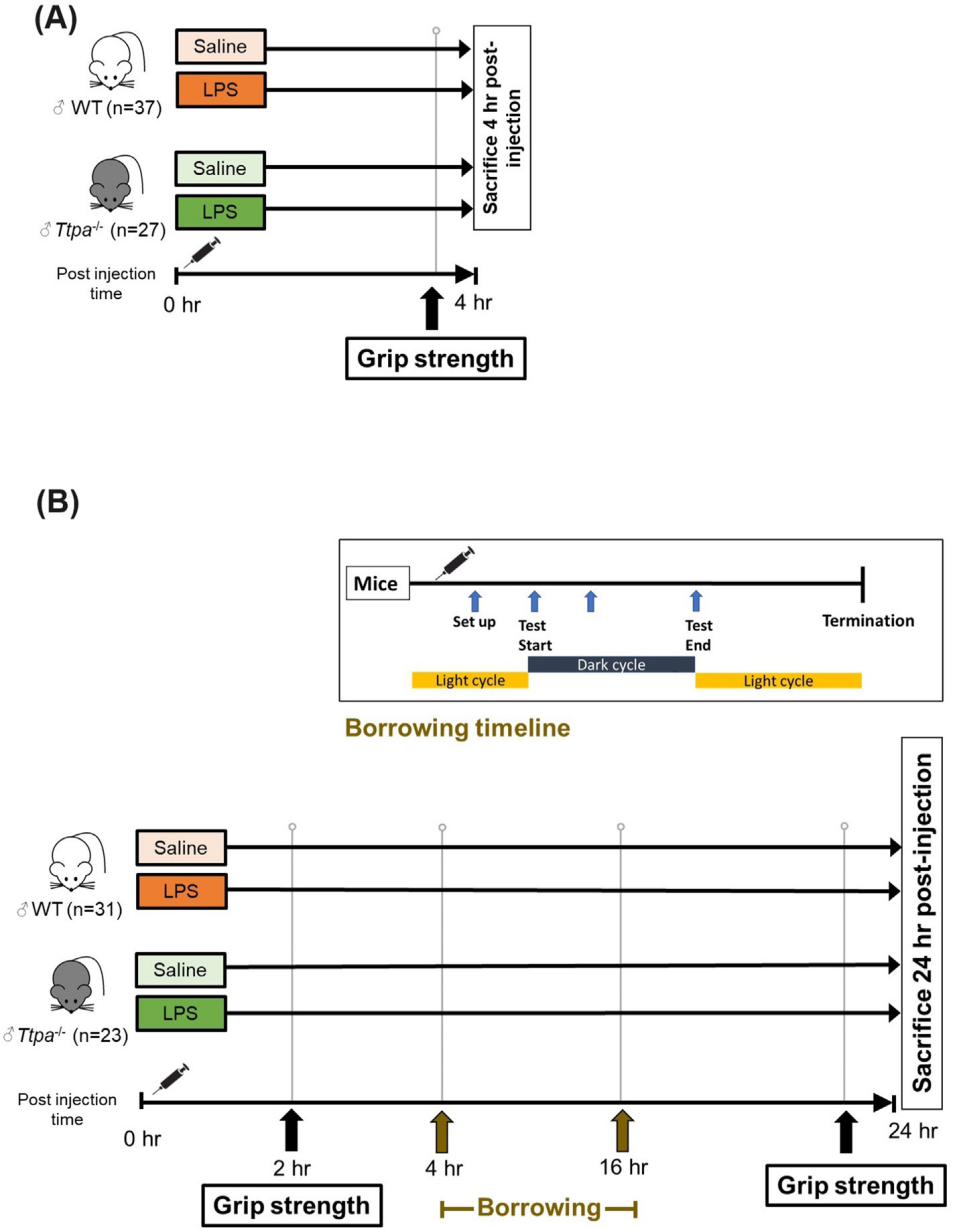
A 12-wk of αT deficiency study design. After 12 wk of an αT-deficient diet, WT, and *Ttpa*^*–/–*^ mice were injected intraperitoneally with LPS (10 μL/mouse) or saline and sacrificed 4 h (Study 1) (A) or 24 h post-injection (Study 2) (B). Before LPS treatment, all study mice underwent behavioral tests as a baseline or training phase. A mouse grip strength test (before/after injection) was conducted for a 4-h post-injection study. Two behavior tests were conducted for a 24-h post-after-injection study. CON, control; *Ttpa*, α-tocopherol transfer protein; WT, wild-type.

The timeline for LPS treatment, behavior tests, and tissue collection is shown in Figure 1. The study design of the 4-h post-injection study (Study 1) was the same as the previously described procedure except for an additional 3-wk-depletion period (Figure 1A) [15]. For the 24-h post-injection study (Study 2) (Figure 1B), mice were injected intraperitoneally with either LPS (10 μg/mouse; Sigma; O127:B8, L4516) or saline as control 24 h before the termination, and the diet was removed from cages in the morning of the termination (8 h fasting period). A 4- or 24-h treatment with LPS was determined based on studies that were similar to our current study designs [[14], [15],[22], [23],^25^]. LPS-induced neuroinflammation was measured 4 h after injection [25], and oxidative stress markers were upregulated in several brain regions [22,25]. Berg et al. [23] showed that LPS treatment 24 h after injection decreased plasma glutathione peroxidase (GPX) activity and observed a trend of plasma αT reduction in WT mice.

As a terminal procedure, the study mice were anesthetized with ketamine/xylazine (87 and 13 mg/mL, respectively) and sacrificed by perfusion with saline (∼10 mL/mouse) per the previously described procedure [15]. In brief, upon reaching a surgical plane of anesthesia, a cardiac puncture was performed, and blood was collected in 10 μL 200-mM EDTA-coated tube. The blood sample tubes were kept on ice for ∼30–60 min. Serum was obtained from blood by centrifugation at 2400 × *g* for 10 min, 4°C using a 5417R Eppendorf centrifuge (F-45-30-11 Rotor, max 30 × 3.75 g). Tissues were dissected, weighed, and immediately snap-frozen in liquid nitrogen. Brain regions were further dissected to isolate the cerebral cortex, cerebellum, and hippocampus.

### Vitamin E analysis in diets and tissues

All experimental diets were custom-designed, and diet preparation procedures were followed those outlined in a previous study [15]. The αT-deficient and LOW diets were purchased from Research Diets, Inc. *RRR*-αTA [Novatol 6–92 (oil); Archer Daniels Midland®] was added to the LOW diet after verifying its purity via HPLC with photodiode array detection (HPLC-PDA). We used HPLC-PDA to determine the αT and αTA concentrations in final diets and vitamin E-containing dietary ingredients (Novatol supplement and soybean oil). The lower limit of detection for αTA in the study diet was 0.49 mg/kg diet. The diets were preserved by vacuum-sealing at –20°C, and fresh pellets were given to the study mice weekly.

Levels of αT in serum and target tissues (brain, liver, arm muscle, and bone marrow stem cells) were analyzed via HPLC-PDA as previously described [29,30]. Briefly, 0.05–0.15 g/tissue or 0.02 g/cell sample was homogenized with ethanol with 0.1% BHT, saturated KOH, and ascorbic acid and saponified for 30 min. αT was extracted 3 times with hexane. Once samples were dried down under argon, they were reconstituted with mobile phase B (8% methanol, 90% MTBE, and 2% ammonium acetate aqueous solution) for HPLC. The wavelength 292 nm was used to detect αT in HPLC. The αT accumulation in adipose tissue was measured with previously described procedures [31]. Adipose tissues (∼0.125 g/sample) were homogenized with phosphate buffered saline (PBS) and chloroform. The lower phase was collected and added to 5.5% ethanol-KOH and 1.2% ethanol-pyrogallol. After saponification, αT was extracted with hexane and analyzed via HPLC. The lower limits of detection for αT were 0.11 μmol/L for serum and 0.12 nmol/g for tissues.

### Mouse grip strength test

The assessment of grip strength is a commonly employed method for examining neuromuscular function in both humans and rodents [32,33]. The Mouse Grip Strength Meter (Columbus Instruments: Chatillion Model DFE-002) was used to examine whether LPS-induced grip weakness was affected by low vitamin E status. All study mice were tested 2 days before the termination (baseline) and ∼3.5 h (4 h post-injection termination) and 23.5 h (24 h post-injection termination) after injection (Figure 1). We also measured grip strength 2 h after LPS treatment to confirm acute inflammatory response in mice at 24-h post-injection study. During the measurements, mice were allowed to hold onto the bar and were then pulled back slowly, and the point of strength at which mice released the bar was determined as maximal muscle strength. For each time point, measurements were repeated 5 times, and these values were averaged to indicate individual mouse grip strength.

#### Borrowing test

Burrowing is a natural rodent behavior that can be measured under controlled laboratory conditions. The procedure was based on previous studies [34,35]. One day before peripheral administration of LPS or vehicle injection (training phase) and after injection (test phase), the burrowing test was conducted to assess sickness behavioral dysfunction. Study mice were tested at the beginning of the dark cycle and 6 and 16 h post-injection (2 and 12 h after dark cycle) (Figure 1B). The plastic tube was 200 mm long and 68 mm in diameter. The open end of the tube was raised 30 mm by bolting two 50 mm machine screws through it, each 10 mm in from the end, spaced just less than a quadrant of the tube apart. A pea gravel-filled burrow tube (∼500 g/tube) [34,35] was placed against the longer wall of a clean cage with a thin layer of bedding, and each mouse was placed in rat-sized cage. Pea gravel, little stones 0.5–1 cm in size, were washed and autoclaved before usage. We weighed the pea gravel in a tube at each study period (at injection, 30 min before the dark cycle, at the dark cycle, 2 and 12 h after the dark cycle) and recorded the amount of material displaced from the burrow. Preliminary testing showed that there was no difference in burrowing between food pellets and pea gravel (data not shown); therefore, we chose to use pea gravel for this study. Mice showing abnormal behavior (for example, showing no interest in burrowing pea gravel overnight) during a training phase were excluded from the data.

### Inflammatory and oxidative stress responses

IL-6 in the heart was measured by ELISA (Mouse IL-6 ELISA Kit RAB0309; Sigma), following the manufacturer’s instructions. The procedures for protein extraction and quantification have been previously described [14]. Briefly, heart samples (∼20 mg) were homogenized with 1 × PBS containing 1% protease inhibitor cocktail (Sigma) and 1% triton × 100 (Sigma) to obtain total protein extracts. Heart protein concentration was analyzed by the Pierce BCA Protein Assay Kit (Thermo Fisher Scientific). All samples were analyzed in duplicate (100–150 μg for heart samples used per well). Concentrations of IL-6 in the heart were determined using a SoftMax Pro 5.2 (Molecular Devices) plate reader.

The expression of genes related to inflammatory and oxidative stress responses (*Il6, Ccl2, Gsr, Gpx1*, etc.) were examined via RT-PCR. Total RNA isolation and cDNA synthesis in the hippocampus and liver were carried out following the manufacturer’s instructions (Thermo Fisher Scientific). Briefly, total RNA in the hippocampus and liver (∼20 mg/sample) was isolated using 750 μL of TRIzol Reagent with a sonicator. cDNA was synthesized from total RNA isolation by a High-Capacity cDNA Reverse Transcription Kit (Thermo Fisher Scientific). RNA purities and concentrations were measured by a Nano-drop 2000 spectrophotometer (Thermo Fisher Scientific) and agarose gel electrophoresis. After all RNA samples were confirmed to meet the quality control requirements, RT-PCR was carried out using a Quant Studio 3 Real-Time PCR system (Applied Biosystems by Thermo Fisher Scientific) according to the manufacturer’s instructions for PowerUp SYBR Green Master Mix (Applied Biosystems). The 2^−ΔΔCt^ method was used to calculate relative gene expression levels. α-Tubulin (5′-CAGGGCTTCTTGGTTTTCC-3′ and 5′-GGTGGTGTGGGTGGTGAG-3′) was used as a reference gene for hippocampal and liver samples.

The procedures for total RNA isolation, cDNA synthesis, quality check, and RT-PCR analysis in the spinal cord have been previously described [14]. All primers were obtained from Integrated DNA Technologies and primer sequences are listed in Supplemental Table 2.

### Circulating immune cell profiles

The circulating immune cell profile was analyzed using an Element HT5 (Heska), following the manufacturer’s instructions. The procedure has been previously described [14]. The device can measure the complexity and granularity of cell volumes and types by triple-angle laser scatter and provide comprehensive immune cell profiles. The number and percent of lymphocytes, monocytes, neutrophils, eosinophils, and basophils were determined to examine the acute immune response induced by LPS and/or low vitamin E status in mice. Briefly, 15–20 μL of each fresh blood per sample was immediately analyzed following a cardiac puncture, and all samples were analyzed in duplicate.

### Statistical analysis

GraphPad Prism version 8.1.3 and G∗Power 3.1.9.7 for Windows were used for statistical analysis. For the assessment of normal distribution and quality of variances, the Shapiro–Wilk and Brown–Forsythe tests were carried out, respectively. In cases where the data did not conform to these assumptions, we applied the following transformations: Y = log(Y) or sqrt(Y). If the assumptions remained unmet even after these transformations, we employed the nonparametric Kruskal–Wallis test. Independent variables were LPS treatment (LPS compared with saline) and genotype (WT compared with *Ttpa*^*–/–*^ mice). Dependent variables were αT concentrations, grip strength, burrowing, gene/protein expression, weight and height, and circulating immune profiles. For most study endpoints, differences between genotype (*Ttpa*^*–/–*^ or WT mice) and treatment groups (LPS 10 μg or saline) were assessed 2 × 2 factorial analysis of variance (ANOVA), followed by Tukey’s post hoc test when justified. If there was no interaction between independent variables, a post hoc test was not conducted. To assess the time, genotype, and LPS treatment difference of behavior tests and weight and height, 3-way repeated Factorial ANOVA, followed by Tukey’s post hoc test, was conducted. Values are represented as mean ± SEM. Differences between experimental groups were considered significant when *P* < 0.05.

## Results

### Study 1: 4-h post-injection study

#### Body mass and food consumption

Weekly body weight was increased over time for all study mice (*P* < 0.0001); however, there was no difference between the genotypes (*P* = 0.10) or treatment groups (*P* = 0.77) (Supplemental Figure 2A). No interactions (week × genotype, week × LPS treatment, genotype × LPS treatment, and week × genotype × LPS treatment) were noted through 3-way repeated measures ANOVA (*P* > 0.10). Along with body weight, average daily food intake was increased for all mice throughout the study period, especially in the first 2–3 wk after weaning (Supplemental Figure 2B). However, there were no main effects (genotype and LPS treatment) or interactions (week × genotype, week × LPS treatment, genotype × LPS treatment, and week ×genotype × LPS treatment) in weekly food consumption among study mice.

#### αT analysis

Accumulation of αT in serum and targeted tissues was examined in WT and *Ttpa*^*–/–*^ mice (*n* = 3–12/group) (Table 1). To assess the effect of LPS treatment or genotype on αT accumulation in tissues at 4 h post-injection, we conducted a 2 × 2 factorial ANOVA. Accumulation of hepatic αT in WT mice was higher than in *Ttpa*^*–/–*^ mice (*P* < 0.0001); however, there was no main effect of LPS treatment (*P* = 0.09) or genotype × LPS treatment interaction (*P* = 0.15) in hepatic αT levels. Concentrations of αT in the adipose tissue of WT mice were higher than in *Ttpa*^*–/–*^ mice (*P* < 0.0001), but there was no difference between LPS and control groups (*P* = 0.27) or genotype × LPS interaction (*P* = 0.24). Brain and serum αT levels in *Ttpa*^*–/–*^ mice were not detectable, while WT mice showed low levels of αT (14.9 ± 0.3 nmol/g and 3.75 ± 0.3 nmol/L, respectively). The Student *t*-test did not show any significant differences in serum and brain αT levels between LPS-treated compared with control WT groups (*P* > 0.98 or 0.65, respectively).

**TABLE 1:** αT concentrations in selected tissues of male *Ttpa*^*–/––/–*^ and WT mice at 4-h post-injection.^1^

	WT mice		*Ttpa*^*–/––/–*^ mice		*P* values		
Tissue	CON	LPS	CON	LPS	Genotype	LPS treatment	Interaction
Liver	12.8 ± 1.0	12.0 ± 0.6	3.2 ± 0.5	2.3 ± 2.8	<0.0001	0.09	0.15
Adipose tissue	23.1 ± 4.4	28.1 ± 2.3	1.3 ± 0.3	1.2 ± 0.2	<0.0001	0.27	0.24
Brain	14.8 ± 0.5	15.1 ± 0.5	ND^2^	ND^2^	—	—	—
Serum	3.7 ± 0.4	3.8 ± 0.6	ND^2^	ND^2^	—	—	—

Abbreviations: ANOVA, analysis of variance; CON, control; LPS, lipopolysaccharide; *Ttpa*, α-tocopherol transfer protein; WT, wild-type.

1 Results in targeted tissues are shown as mean ± SEM nmol/g or μmol/L (*n* = 3–12/treatment group). 2 × 2 factorial ANOVA was conducted to measure a difference in the accumulation of αT in tissues or serum between genotypes and treatment groups.

2 ND, lower limits of detection: 0.12 nmol/g (tissues) and 0.11 μmol/L.

#### Sickness behavior test

**FIGURE 2:**
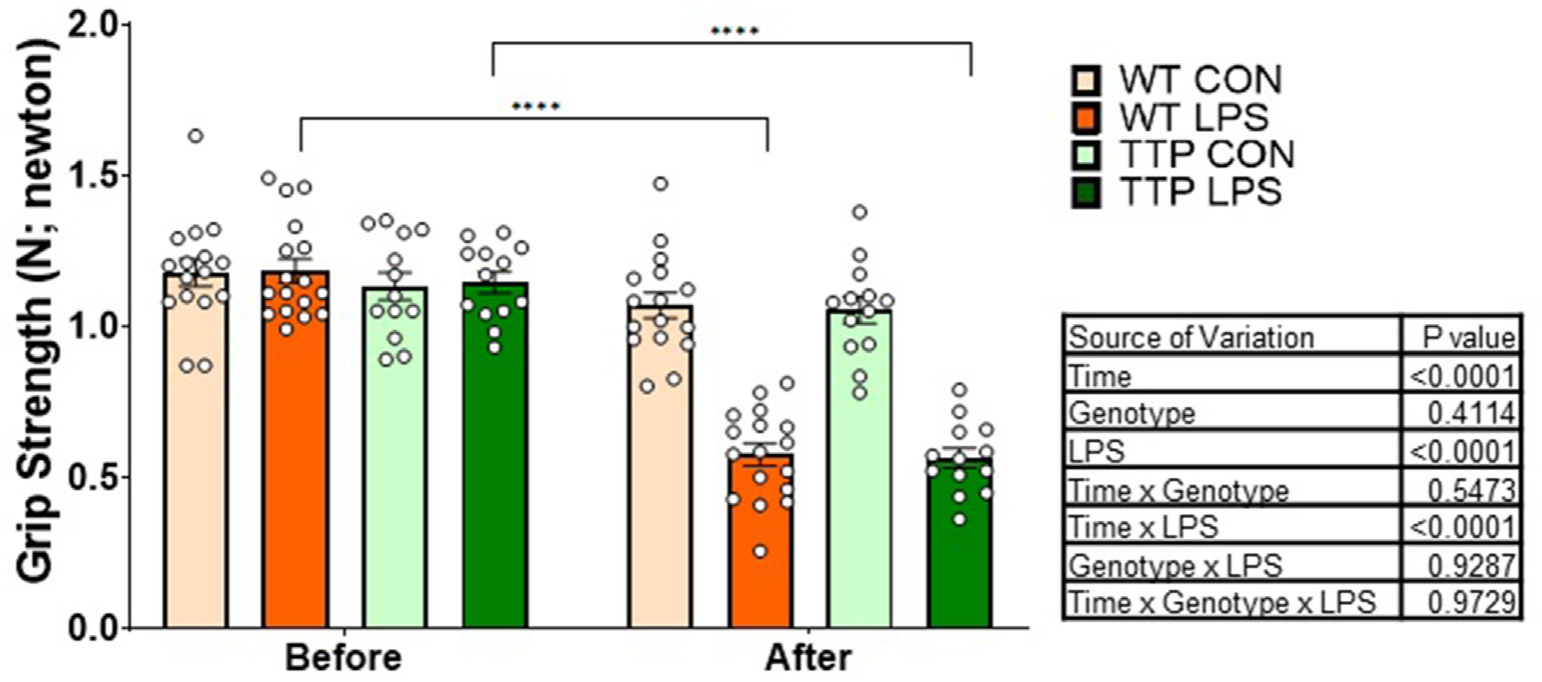
Effect of vitamin E deficiency and LPS injection on grip strength at 4 h after injection. Mouse grip strength test was conducted in *Ttpa*^*–/–*^ and WT mice at 4 h after injection (*n* = 13–19/group). Values are expressed as mean ± SEM (*n* = 13–19/treatment group). A 3-way repeated measures ANOVA evaluation showed the main effect of LPS and Time after injection. However, there was no significant main effect of genotype or any interactions except for a time × LPS interaction. ANOVA, analysis of variance; CON, control; *Ttpa*, α-tocopherol transfer protein; WT, wild-type.

Grip strength tests were conducted in WT and *Ttpa*^*–/–*^ mice 2 days before the termination (baseline) and 3.5 h after injection of LPS or saline (Figure 2). Through 3-way repeated measures ANOVA evaluation, we confirmed that there was no difference in mouse grip strength before LPS injection among treatment groups at baseline (before). After LPS/saline injection, the ANOVA evaluation of grip strength indicated significant main effects of LPS (*P* < 0.0001) and time (baseline compared with 4 h) (*P* < 0.0001) in reducing strength but no significant genotype effect (*P* = 0.41) or interactions (time × genotype, genotype × LPS, or time × genotype × LPS). There was no difference in grip strength of the saline control group between before and after treatment.

#### Acute inflammatory response

To examine whether αT deficiency altered LPS-induced inflammatory responses, IL-6 level in the heart via ELISA and expression of genes related to inflammatory and indirect oxidative stress responses in the hippocampus via RT-PCR were measured. Heart IL-6 level was increased in LPS groups compared with control groups (*P* < 0.0001) (Supplemental Figure 3). ANOVA evaluation showed the main effect of genotype (*P* = 0.03) between control WT and Ttpa^*–/–*^ mice but not a genotype × LPS interaction (*P* = 0.29) in the concentration of heart IL-6.

**FIGURE 3:**
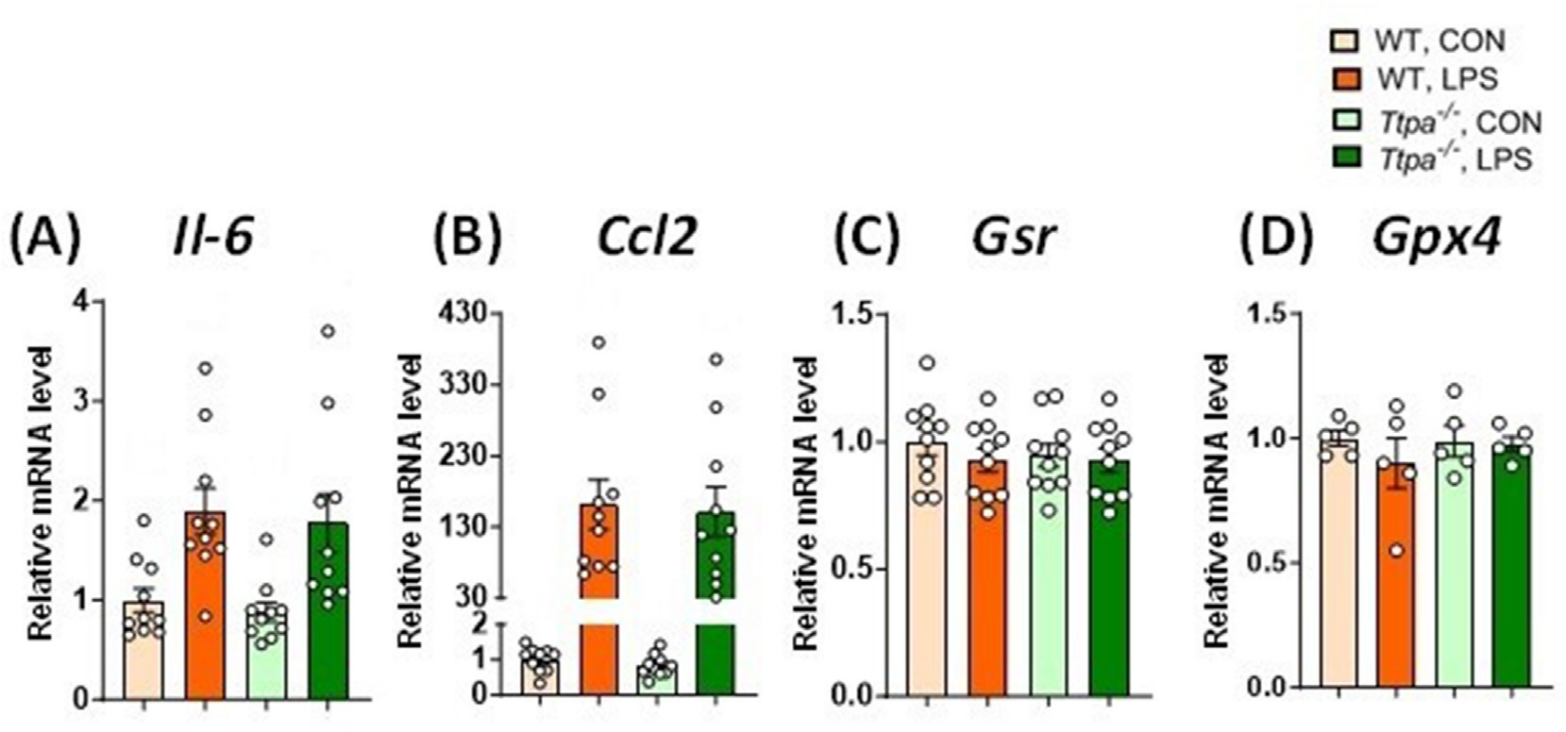
Hippocampal gene expression at 4 h after injection. Hippocampal (A) *Il6*, (B) *Ccl2*, (C) *Gsr*, and (D) *Gpx4* expression of WT and *Ttpa*^*–/–*^ mice at 4 h after injection. Results are shown as mean ± SEMs (*n* = 5–10/group). Italic markers are shown as gene expressions. Expression of genes related to inflammatory response was upregulated by LPS, assessed by 2 × 2 factorial ANOVA. There was no difference in the expression of genes related to indirect oxidative stress responses between LPS treatment groups or genotypes. ANOVA, analysis of variance; CON, control; ND, not detected; *Ttpa*, α-tocopherol transfer protein; WT, wild-type.

To evaluate neuroinflammatory response in the study mice at 4-h post-LPS injection, expression of genes related to inflammatory (for example, *Il6*, *Ccl2*) and indirect oxidative stress responses (for example, *Gsr, Gpx4*) were measured in the hippocampus (Figure 3). Throughout 2 × 2 Factorial ANOVA evaluation, hippocampal *Il6* (Figure 3A) and *Ccl2* (Figure 3B) expression were higher in LPS groups than in control groups (*P* < 0.0001), but there was no main effect of genotype (*P* > 0.39) or genotype × LPS treatment interaction (*P* > 0.92). There was no change in *Gsr* (Figure 3C) or *Gpx4* (Figure 3D) expression between genotype (*P* > 0.58) or LPS treatment (*P* > 0.36).

Complete blood counts and percentages of immune cells were assessed from whole blood via the Heska apparatus (Table 2 and Supplemental Table 3). White blood cells, particularly lymphocytes and monocytes, were decreased 4 h after LPS treatment compared with the saline control groups (Table 2) (*P* < 0.0001). The decrease in lymphocytes and monocytes was reflected in the decreased total white blood cells within LPS groups. A 2× 2 factorial ANOVA evaluation of white blood cells/lymphocyte count indicated significant main effects of LPS (*P* < 0.0001), but not genotype (*P* > 0.92) or a genotype × LPS interaction (*P* > 0.39). Although eosinophils count tended to be decreased by LPS (*P* = 0.07), the number of neutrophils, eosinophils, and basophils in blood did not differ by LPS exposure or genotypes at 4 h post-LPS injection. The proportion of neutrophils increased in LPS groups, while the percentage of lymphocytes decreased (Supplemental Table 3). We did not observe a difference in red blood cells among treatment groups (data not shown).

**TABLE 2:** Blood immune cell levels in *Ttpa*^*–/––/–*^ and WT mice 4 h after injection of LPS or saline.

	WT		*Ttpa*^*–/–*^		*P* values		
Cell type	CON	LPS	CON	LPS	Genotype	LPS treatment	Interaction
WBCs	2.39 ± 0.15	1.07 ± 0.06	2.47 ± 0.17	1.00 ± 0.07	0.92	<0.0001	0.53
Neutrophils	0.51 ± 0.05	0.42 ± 0.03	0.47 ± 0.04	0.40 ± 0.04	0.52	0.14	0.87
Lymphocytes	1.72 ± 0.12	0.54 ± 0.04	1.84 ± 0.14	0.46 ± 0.02	0.94	<0.0001	0.39
Monocytes	0.10 ± 0.01	0.03 ± 0.00	0.10 ± 0.01	0.05 ± 0.01	0.59	<0.0001	0.59
Eosinophils	0.05 ± 0.01	0.07 ± 0.01	0.05 ± 0.01	0.07 ± 0.01	0.79	0.07	0.75
Basophils	0.01 ± 0.00	0.02 ± 0.00	0.01 ± 0.00	0.02 ± 0.00	0.28	0.74	0.35

Abbreviations: ANOVA, analysis of variance; CON, control; LPS, lipopolysaccharide; *Ttpa*, α-tocopherol transfer protein; WBCs, white blood cells; WT, wild-type.

Each value is shown as mean ± SEM 10^3^/μL (*n* = 13–19/group). A 2 × 2 Factorial ANOVA was conducted to examine the difference between genotypes and treatment groups.

### Study 2: 24-h post-injection study

#### Body mass and food consumption

Weekly body weight or average daily food intake was increased in all study mice throughout the study period (*P* < 0.0001). There was no difference in body mass and food intake among study mice per week (*P* > 0.998 and *P* > 0.24, respectively) (Supplemental Figure 4A and B). No main effects of LPS/genotype or interactions were shown in weekly body weight gain and average food consumption throughout 3-way repeated measures ANOVA evaluation.

Comparing before and after LPS/saline injection, while body mass was not significantly changed between the treatment groups (*P* = 0.12) or genotypes (*P* = 0.19), a genotype × LPS treatment interaction showed a difference (*P* = 0.04) by a 2 × 2 factorial ANOVA with Tukey’s multiple comparisons test (data not shown). There were main effects of LPS/genotype and interaction in food consumption before/after injection (*P* > 0.0001). LPS treatment decreased food consumption in both LPS-treated groups compared with the control groups.

#### αT analysis

A 2 × 2 factorial ANOVA evaluation showed that concentrations of αT in the liver and adipose tissue of WT mice were higher than *Ttpa*^*–/–*^ mice (*P* < 0.0001) (Table 3). Furthermore, there was a main effect of LPS treatment (*P* < 0.0001) on the accumulation of αT in the liver. However, there was no genotype × LPS treatment interaction in hepatic αT level (*P* = 0.47). We did not observe a difference in the accumulation of αT in the adipose tissue between LPS and saline control groups (*P* = 0.26) nor a significant interaction (genotype × LPS treatment) in the concentration of αT in the adipose tissue (*P* = 0.12). Concentrations of αT were detected in the arm muscle, bone marrow stem cells, brain, and serum of WT mice (4.8 ± 0.7, 10.6 ± 1.0, and 16.7 ± 0.9 nmol/g, and 4.9 ± 0.2 μmol/L, respectively), but not in these tissues or serum in *Ttpa*^*–/–*^ mice.

**TABLE 3:** αT concentrations in selected tissues of male *Ttpa*^*–/––/–*^ and WT mice at 24-h post-injection.^1^

	WT mice		*Ttpa*^*–/––/–*^ mice		*P* values		
Tissue	CON	LPS	CON	LPS	Genotype	LPS treatment	Interaction
Liver	12.9 ± 0.6	10.2 ± 0.6	5.5 ± 0.7	2.0 ± 0.3	<0.0001	<0.0001	0.47
Adipose tissue	30.7 ± 2.3	25.5 ± 3.3	1.1 ± 0.3	1.9 ± 0.6	<0.0001	0.26	0.12
Brain	15.3 ± 1.1	18.1 ± 0.03	ND^2^	ND^2^	—	—	—
Serum	4.9 ± 0.3	4.8 ± 0.4	ND^2^	ND^2^	—	—	—
Arm muscle	4.1 ± 1.1	5.6 ± 0.7	ND^2^	ND^2^	—	—	—
Bone marrow cells	10.8 ± 1.7	10.3 ± 0.9	ND^2^	ND^2^	—	—	—

Abbreviations: ANOVA, Analysis of Variance; CON, control; LPS, lipopolysaccharide; *Ttpa*, α-tocopherol transfer protein; WT, wild-type.

1 Results in targeted tissues are shown as mean ± SEM nmol/g or μmol/L (*n* = 3–12/treatment group). For the liver, different superscript letters denote significant differences (*P* < 0.0001) between LPS treatment groups and genotypes by 2 × 2 factorial ANOVA.

2 ND, lower limits of detection: 0.12 nmol/g (tissues) and 0.11 μmol/L.

#### Sickness behavior test

**FIGURE 4:**
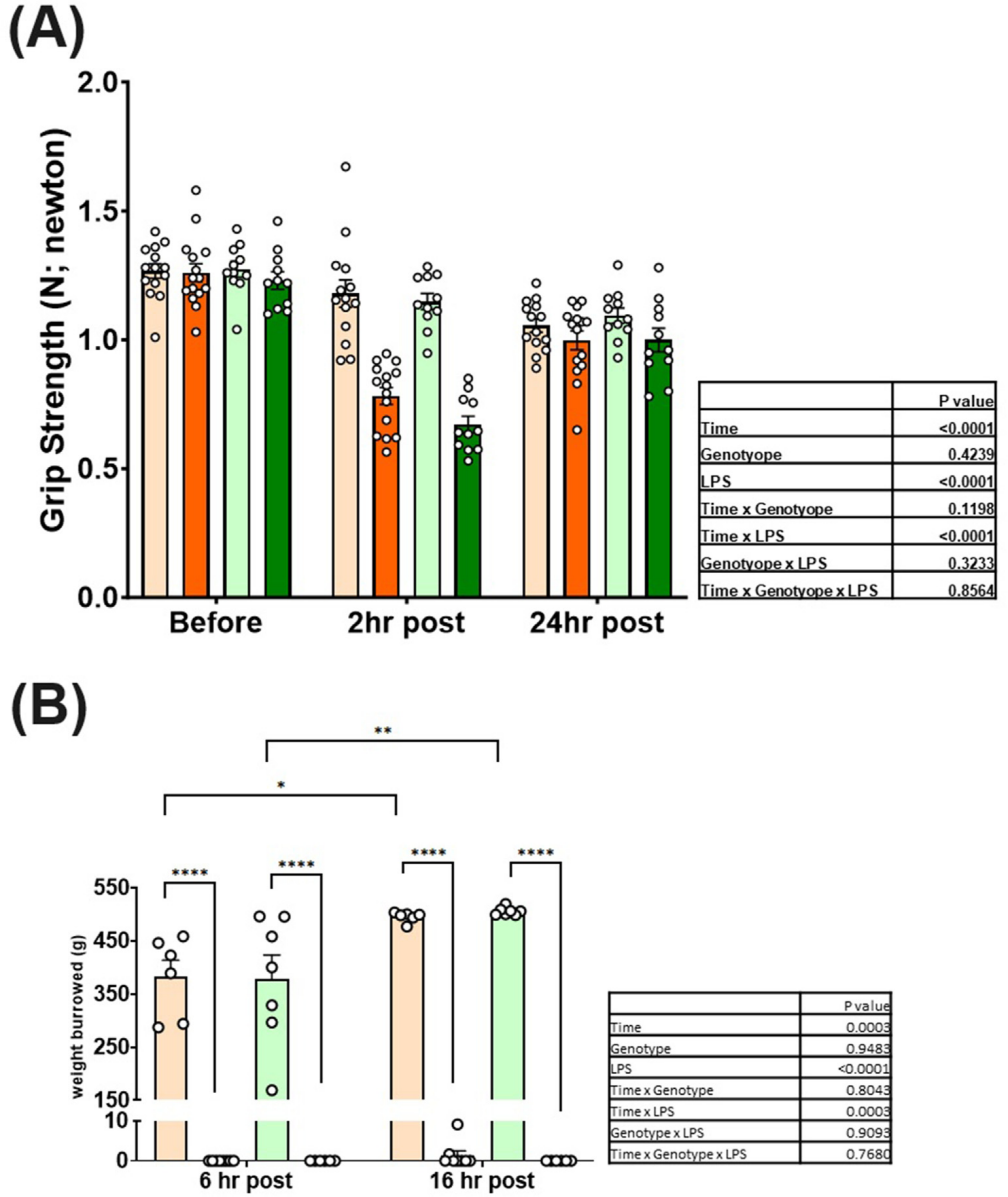
Effect of vitamin E deficiency and LPS injection on grip strength and burrowing at 24 h after injection. Mouse grip strength (*n* = 11–15/group) (A) and burrowing behavior (*n* = 6–9/group) (B) tests were conducted in *Ttpa*^*–/–*^ and WT mice at 24 h after injection. Values are expressed as mean ± SEM. A 3-way repeated measures ANOVA was conducted. ANOVA, analysis of variance; CON, control; *Ttpa*, α-tocopherol transfer protein; WT, wild-type.

A 3-way repeated measures ANOVA was conducted to test the impact of LPS treatment (saline compared with LPS), time (baseline, 2 and 24 h), and genotypes (WT compared with *Ttpa*^*–/–*^ mice). There was a significant time effect resulting in an overall decrease in grip strength compared with baseline (*P* < 0.0001). ANOVA evaluation of grip strength indicated significant main effects of LPS (*P* < 0.0001) and a time × LPS interaction (*P* < 0.0001) but not genotype (*P* = 0.42) or other interactions (Figure 4A). Before injection (baseline), there was no difference in grip strength among the study mice. Furthermore, we found that there was no difference among the study mice 24 h after LPS injection. At 2 h post-injection, LPS groups had lower grip strength compared with control groups (*P* < 0.0001); however, there was no genotype effect between LPS-treated WT and *Ttpa*^*–/–*^ mice. There was a significant difference in grip between 2 and 24 h post-injection (*P* < 0.0001). The grip strength in LPS-treated mice was slightly but significantly lower 24 h after the LPS injection compared with the baseline values (*P* < 0.05).

To further assess whether LPS-induced sickness behavior was exacerbated by αT deficiency, we used a burrowing test. This test was employed during the dark cycle because mice are most active and show burrowing behavior during this period [35,36]. In the training phase, mice removed most pea gravel from a tube 2 h after the beginning of the dark cycle and completely removed all pea gravel from the tube 12 h after the dark cycle (data not shown). However, after the LPS administration, LPS groups did not burrow pea gravel from a tube during any study period, indicating that LPS-induced sickness behavior persisted for ≥16 h post-injection (Figure 4B). ANOVA evaluation of burrowing behavior indicated significant main effects of LPS (*P* < 0.0001) and time (*P* = 0.0003), and a time × LPS interaction (*P* = 0.0003) but not a genotype (*P* = 0.95) effect or the other interactions.

#### Inflammatory and indirect oxidative stress responses

**FIGURE 5:**
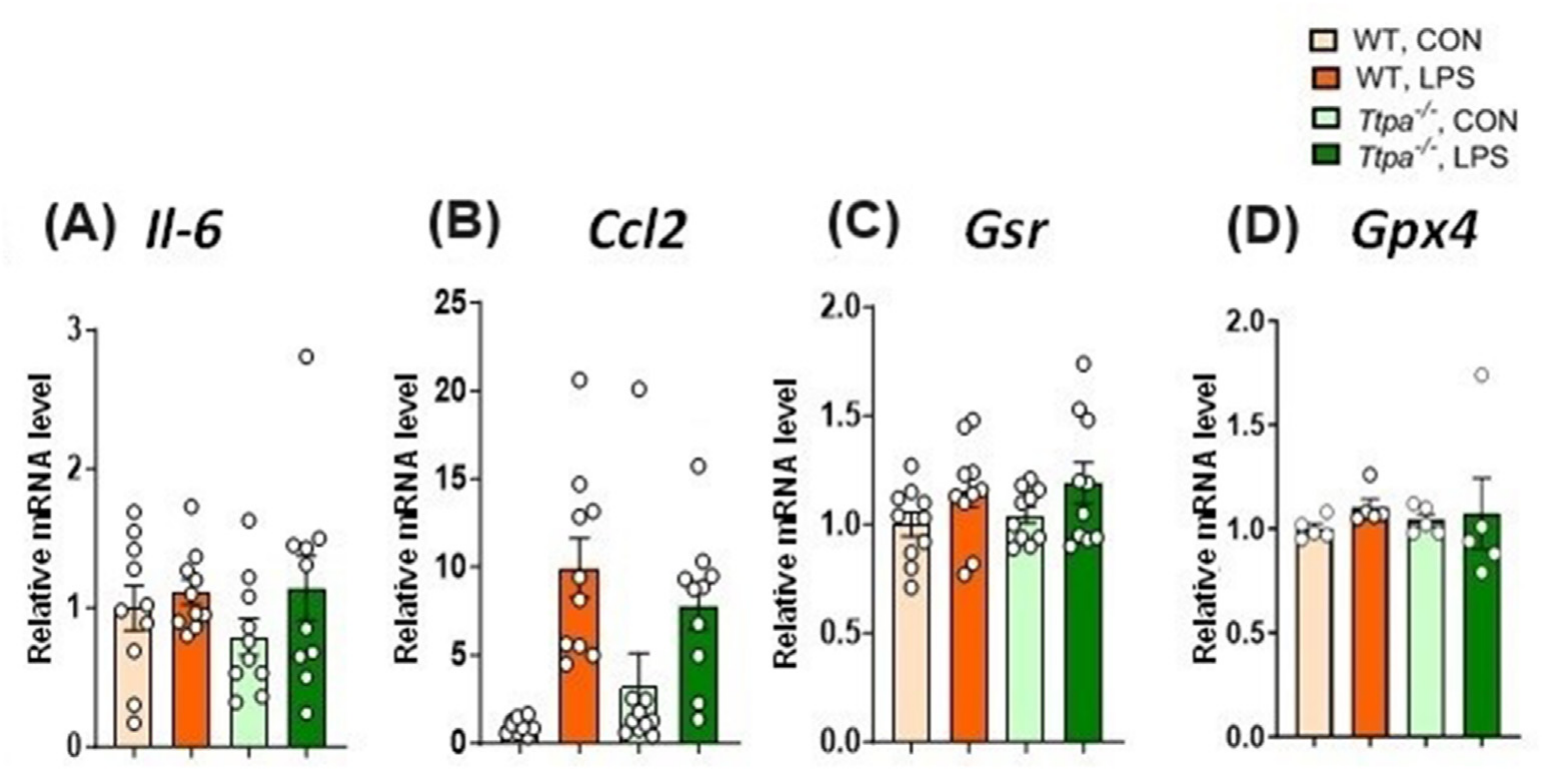
Hippocampal gene expression after 24 h after injection. Hippocampal (A) *Il6*, (B) *Ccl2*, (C) *Gsr*, and (D) *Gpx4* expression of WT and *Ttpa*^*–/–*^ mice at 24 h after injection. Results are shown as mean ± SEMs (*n* = 5–10/group). Italic markers are shown as gene expressions. *Ccl2* and *Gsr* expression was upregulated by LPS, assessed by 2 × 2 factorial ANOVA, while *Il6* and *Gpx4* expression was not changed at 24 h after LPS injection. There was no difference in the expression of genes related to oxidative stress responses between genotypes. No significant genotype × LPS interaction was observed in any gene expression. ANOVA, analysis of variance; CON, control; ND, not detected; *Ttpa*, α-tocopherol transfer protein; WT, wild-type.

**FIGURE 6:**
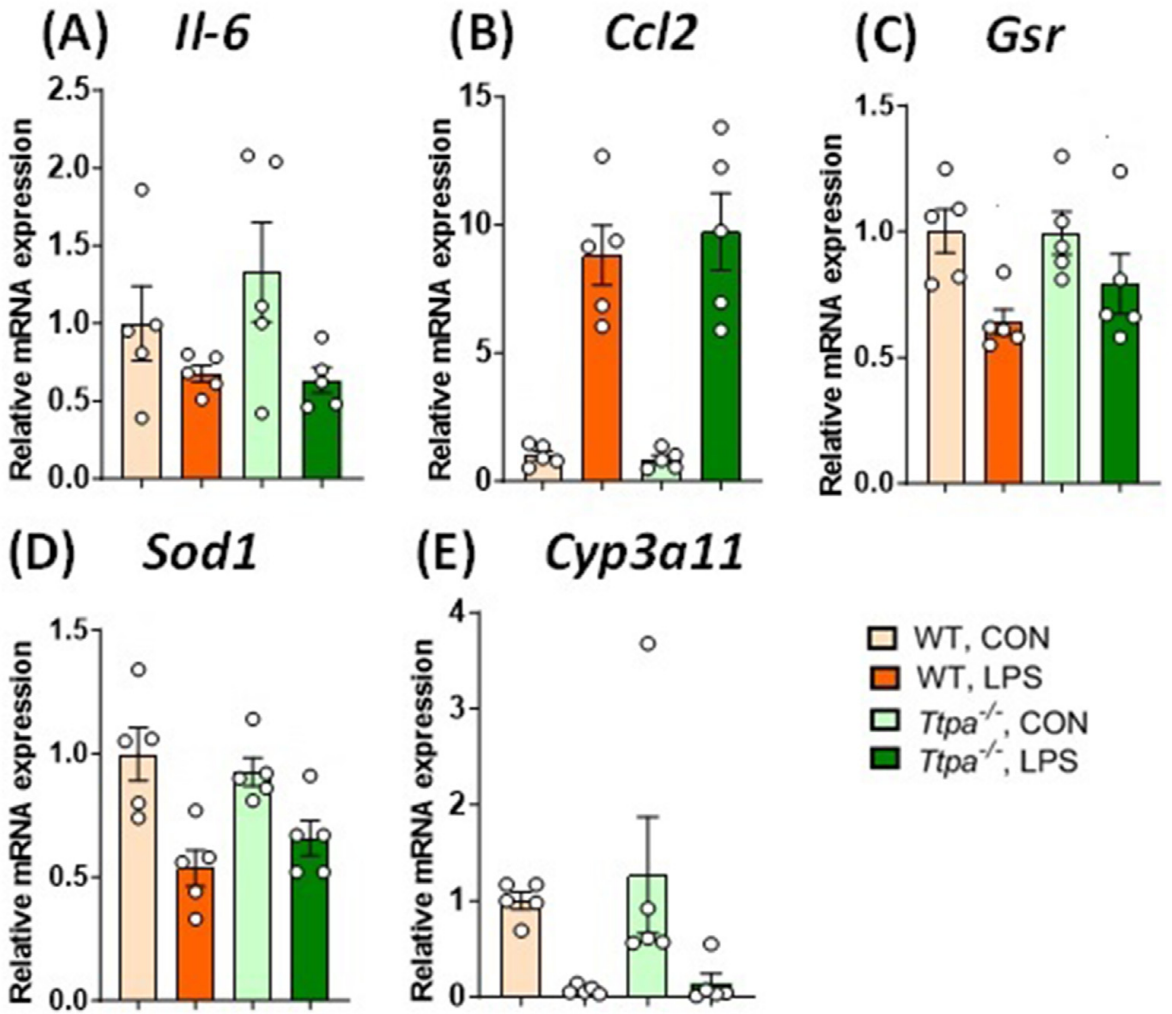
Hepatic gene expression after 24 h after injection. Hepatic (A) *Il6*, (B) *Ccl2*, (C) *Gsr*, (D) *Sod1*, and (E) *Cyp3a11* expression of WT and *Ttpa*^*–/–*^ mice at 24 h post-injection. Results are shown as mean ± SEMs (*n* = 4–5/group). Italic markers are shown as gene expressions. Expression of genes related to inflammatory response was altered by LPS, assessed by 2 × 2 factorial ANOVA or nonparametric Kruskal–Wallis test. ANOVA, analysis of variance; CON, control; ND, not detected; *Ttpa*, α-tocopherol transfer protein; WT, wild-type.

To examine whether αT deficiency altered LPS-induced inflammatory response at 24 h post-injection, expression of genes, such as *Il6, Gsr,* and *Ccl2,* in the hippocampus, spinal cord, and liver of WT and *Ttpa*^*–/–*^ mice were measured via RT-PCR (FIGURE 5, FIGURE 6). Hippocampal *Il6* (Figure 5A) and *Gpx4* (Figure 5E) gene expression was not changed either by LPS or genotype. *Gsr* (Figure 5C) and *Ccl2* (Figure 5B) expression in the hippocampus was upregulated by LPS (*P* < 0.05), but we did not find any differences in the main effect of genotype or a genotype × LPS interaction. There was a slight trend of a genotype × LPS interaction in *Ccl2* expression 24 h after LPS/saline injection (*P* = 0.08). In the spinal cord, there were no main effects of genotype or LPS or no interaction in *Il6* and *Gpx1* expression (data not shown).

To further probe the impact of LPS treatment in the liver, based on the reduction of hepatic αT 24 h after injection, we measured markers of inflammatory and indirect oxidative stress responses and enzymes related to vitamin E turnover via RT-PCR (Figure 6). A 2 × 2 factorial ANOVA or nonparametric Kruskal–Wallis evaluation showed that LPS-treated mice had downregulated *Il6* (Figure 6A), *Gsr* (Figure 6C)*, Sod1* (Figure 6D), and *Cyp3a11* (Figure 6E) and upregulated *Ccl2* (Figure 6B) expression at 24 h post-injection (*P* < 0.05), indicating a significant main effect of LPS treatment. However, there was no main effect of genotype or a genotype × LPS interaction in any of the gene expression. Overall, there was no difference in these markers between WT and *Ttpa*^*–/–*^ mice.

To determine whether αT deficiency altered 24 h post-injection immune cell profiles in blood, we measured complete blood counts from whole blood. White blood cells, particularly lymphocytes, were decreased in LPS groups 24 h after LPS treatment compared with the control groups (*P* < 0.0001) (Table 4). The decrease in lymphocytes was reflected in the total white blood cells within LPS groups. The number of monocytes and eosinophils in blood did not differ by LPS exposure or genotypes. Basophils were not affected by LPS, but there was a trend of genotype (*P* = 0.08). Basophils were lower in *Ttpa*^*–/–*^ mice than WT mice. Neutrophils were increased by LPS *(P* = 0.001). Interestingly, a 2 × 2 factorial ANOVA evaluation indicated a main effect of genotype (*P* < 0.01). Although there was no significant interaction of LPS × genotype, we observed that control *Ttpa*^*–/–*^ mice had decreased white blood cells and lymphocytes compared with control WT mice. The proportion of neutrophils increased in LPS groups, while the percentage of lymphocytes decreased (Supplemental Table 4). We did not observe a difference in red blood cells among treatment groups (data not shown).

**TABLE 4:** Blood immune cell levels in *Ttpa*^*–/––/–*^ and WT mice at 24 h after injection of LPS or saline.

	WT	*Ttpa*^*–/–*^			*P* values		
Cell type	CON	LPS	CON	LPS	Genotype	LPS treatment	Interaction
WBCs	5.26 ± 0.32	2.02 ± 0.12	3.51 ± 0.21	1.61 ± 0.10	0.002	<0.0001	0.22
Neutrophils	0.79 ± 0.05	1.31 ± 0.12	0.55 ± 0.04	0.98 ± 0.10	0.17	0.001	0.70
Lymphocytes	3.91 ± 0.28	0.50 ± 0.07	2.83 ± 0.20	0.42 ± 0.04	0.008	<0.0001	0.15
Monocytes	0.16 ± 0.01	0.08 ± 0.01	0.07 ± 0.01	0.08 ± 0.01	0.14	0.24	0.20
Eosinophils	0.26 ± 0.06	0.06 ± 0.01	0.04 ± 0.01	0.10 ± 0.01	0.08	0.52	0.05
Basophils	0.15 ± 0.02	0.06 ± 0.03	0.01 ± 0.00	0.04 ± 0.00	0.08	0.46	0.86

Abbreviations: ANOVA, analysis of variance; CON, control; LPS, lipopolysaccharide; *Ttpa*, α-tocopherol transfer protein; WBCs, white blood cells; WT, wild-type.

Each value is shown as mean ± SEM 10^3^/μL (*n* = 10–13/group). A 2 × 2 Factorial ANOVA was conducted to examine the difference between genotypes or treatment groups.

## Discussion

The present study was designed to assess whether 12 wk of an αT-deficient diet differentially affected LPS-induced inflammatory responses between WT and *Ttpa*^*–/–*^ mice at 4 h after injection (Study 1) and 24 h post-injection (Study 2). The summary table of the impact of LPS/genotypes by time point is shown in Table 5. In Study 1, LPS-treated mice showed increased proinflammatory cytokine production and reduced grip strength, indicating acute inflammatory responses. However, there was no significant difference in any targeted markers between WT and *Ttpa*^*–/–*^ mice. In Study 2, LPS-induced inflammatory responses remained but there was partially recovered at 24-h post-injection. Surprisingly, LPS dosing further reduced hepatic αT levels 24 h after LPS injection (discussed ahead). However, we did not observe any further differences in any LPS-induced changes between the genotypes. We confirmed that the LPS administration caused sickness behavior and increased inflammatory levels. For most endpoints, mice were more severely impacted 4 h after injection and showed partial acute/recovery inflammatory responses by 24 h post-LPS dose.

**TABLE 5:** Summary of the impact of LPS/genotype on outcome/endpoints of this study.

Outcome/endpoints	Study 1		Study 2	
	4 h after injection		24 h after injection	
	LPS	Genotype	LPS	Genotype
Weekly body mass change	—	—	—	—
Weekly food consumption	—	—	—1	—1
Concentration of αT				
Liver	—	*P* < 0.0001	*P* < 0.0001	*P* < 0.0001
Adipose tissue	—	*P* < 0.0001	—	*P* < 0.0001
Serum	—	Undetectable	—	Undetectable
Brain	—	Undetectable	—	Undetectable
Hippocampal mRNA expression level				
*Il-6*	*P* < 0.0001	—	—	—
*Ccl2*	*P* < 0.0001	—	*P* < 0.05	—
*Gpx4*	—	—	—	—
*Gsr*	—	—	*P* < 0.05	—
Hepatic mRNA expression level^2^				
*Il-6*	—		*P* < 0.05	—
*Ccl2*	—		*P* < 0.0001	—
*Gsr*	—		*P* < 0.01	—
*Sod1*	—		*P* < 0.001	—
*Cyp3a11*	—		*P* < 0.01	—
Grip strength test				
Grip strength test	*P* < 0.0001	—	*P* < 0.0001	—
Complete blood counts				
White blood cells	*P* < 0.0001	—	*P* < 0.0001	*P* < 0.01
Neutrophils	—	—	*P* = 0.001	—
Lymphocytes	*P* < 0.0001	—	*P* < 0.0001	*P* < 0.01
Monocytes	*P* < 0.0001	—	—	—
Eosinophils	—	—	—	*P* = 0.08
Basophils	—	—	—	*P* = 0.08

Abbreviation: LPS, lipopolysaccharide.

1 Weekly food consumption was not altered by LPS/genotype. However, comparing before and after LPS injection, there was a difference in food consumption between LPS groups and these genotypes.

2 Hepatic mRNA levels were only measured at 24 h after LPS injection.

In Study 1, LPS administration caused proinflammatory cytokine production and sickness behaviors in mice 4 h after injection, indicating acute inflammatory response in LPS-treated mice. Previous studies from other laboratories have similarly reported peak proinflammatory cytokine production and sickness behavior occurring 2–4 h after LPS treatment [20,23,24,37]. The immune cell levels in whole blood 4 h post-injection in this study were consistent with the levels observed in our previous studies [14,15]. Although we confirmed the acute inflammatory response, we did not observe a difference in the expression of genes related indirectly to oxidative stress in the hippocampus or any changes of body weight or food intake in the treatment group.

According to previous literature, LPS administration causes relatively rapid oxidative stress responses in murine brains. A single dose of intraperitoneal LPS [1 mg/kg body weight (BW)] injection in male C57BL/6 mice (13 wk old) decreased glutathione-disulfide reductase (GSR) activity in the brain 3 and 48 h after LPS administration, resulting in a lower ratio of glutathione (GSH)/glutathione disulfide [38]. Another study indicated that adult male C57BL/6NHsd mice had a significant reduction in GSH level 12 h after an intraperitoneal LPS injection (*Escherichia coli* 0111:B4, 6.75 × 10^4^ endotoxin unit/g BW ≈ 6.75–67.5 mg/kg BW), suggesting decreased GSR activity [39]. In our study, the expression of genes related to inflammation and genes related to an indirect oxidative stress response in the brain were altered by LPS dependent upon the time of sacrifice. We confirmed that LPS groups upregulated hippocampal *Il6* and *Ccl2* expression at 4 h post-injection but not *Gsr* or *Gpx4* expression. Furthermore, we did not find a difference in those markers of the brain by genotype.

We previously fed mice with αT-depleted diets for 4 and 9 wk and found that these feeding periods resulted in a trend (n.s.) of the genotype effects following LPS-induced acute inflammatory stress response [14,15]. At 9 wk of αT deficiency, grip strength was reduced in LPS-treated groups, an effect that was more pronounced in *Ttpa*^*–/–*^ mice [15]. Surprisingly, we did not find genotype differences in grip strength in this study following 12 wk of αT deficiency. This could be due to low power for the sample size (*β* = 0.78). It is also possible that 12 wk of an αT-deficient diet may have been too long to tease out differences between WT and *Ttpa*^*–/–*^ mice; perhaps both genotypes were so depleted that detection of genotype differences was not possible. Using WT mice with a standard, αT containing diet as a positive control, might be needed to establish the most appropriate αT depletion study period.

In Study 2, study mice had some acute inflammatory responses but there was partially recovered 24 h after LPS injection. We observed that LPS administration resulted in changes, such as decreased food intake and altered behaviors (Figure 4), possibly due to sickness/fatigue. Body weight changes were noted 8 h after injection in LPS-treated mice [22]. However, we did not observe a change in body weight before and after LPS dosing at 24 h post-injection. We observed a difference in food intake between LPS and saline groups at 24 h post-LPS dose.

We found that at 24 h post-injection, the concentration of αT in the liver was decreased by LPS even though the study mice had very low αT status, suggesting a direct impact of LPS on hepatic αT storage/turnover. Compared with our finding, Berg et al. [23] observed a trend of plasma αT reduction 24 h after LPS (1 μg/mouse) in WT mice. These researchers also reported that only high αT supplemented (500 mg/kg diet) mice had a significant decrease of hepatic αT 24 h after LPS administration but not in the low or adequate αT supplemented groups (10 and 75 mg/kg diet, respectively) [23]. It is possible that LPS impacted the turnover of hepatic αT due to increased inflammation, as indicated by increased *Ccl2*, and/or altered synthesis of enzymes related to inflammatory responses, such as noted with changes in hepatic *Cyp3a11*, *Sod1*, and *Gsr*. *Cyp3a11* is a marker of αT turnover, being upregulated with excessive αT accumulation in the body and downregulated with LPS challenge-induced oxidative stress [40]. We confirmed LPS-induced inflammatory responses in the liver with upregulated *Ccl2* and downregulated *Cyp3a11*, *Sod1*, and *Gsr*, respectively. However, surprisingly, we observed downregulated *Il6* in the liver 24 h after LPS injection, while hippocampal and spinal cord *Il6* were not changed between LPS and control groups. This observation could be because the liver was the initial/rapid responder to intraperitoneal LPS injection with IL-6 secretion into the circulation for delivery to extrahepatic tissues.

We also found upregulated hippocampal *Gsr* expression at 24 h post-LPS administration, suggesting an indirect oxidative stress response 24 h after LPS treatment. The production of ROS, such as superoxide radicals, is modulated by superoxide dismutase, catalase, and GPX [41]. This process is further exacerbated by immune cell activation and inflammation. One previous study showed that LPS-treated mice (1 μg/mouse) had decreased plasma GPX activity assessed by a kinetic colorimetric assay 24 h after injection, while vitamin E supplementation improved its activity [23]. Based on the literature, we hypothesized that *Gsr* and *Gpx* expression in the brain would be decreased by LPS and/or low vitamin E status; however, we did not observe the impact of low vitamin E status on neuroinflammatory responses after LPS dosing. We did not directly measure oxidative stress levels, such as 8-isoprostane or malondialdehyde. These biomarkers should be measured for future studies to assess oxidative stress levels. Overall, we confirmed the impact of LPS at the different time periods and observed the initial indirect oxidative stress response in the brain 24 h after LPS injection.

In terms of sickness behaviors, at 16 h post-injection (12 h post-dark cycle), LPS-treated mice did not remove any pea gravel during the burrowing test, a finding that was previously noted [20]. Teeling et al. [20] reported substantial recovery of burrowing behavior overnight, while our study mice did not recover from this sickness behavior. That may be due to the higher dose we used (∼0.43 mg/kg BW) compared with their lower dose range (0–100 μg/kg BW). There was a difference in mouse grip strength between LPS and control groups 2 h after injection, but the LPS groups almost fully recovered their grip strength 24 h after LPS treatment. In our prior study of 9-wk depleted mice, grip strength in *Ttpa*^*–/–*^ mice was further reduced at 4 h after injection compared with WT mice, but there was no difference in any targeted biomarkers of inflammatory and oxidative stress responses between WT and *Ttpa*^*–/–*^ mice. [15]. In this study, the grip strength of study mice at 24 h post-injection was also measured at 2 h post-injection. However, there was no difference between LPS-treated WT and *Ttpa*^*–/–*^ mice 2 and 24 h after injection.

Compared with Study 1 and our previous studies [14,15], Study 2 demonstrated that circulating immune cells differed at 24 h post-injection. Monocyte levels recovered and neutrophils in blood were increased 24 h after injection. We confirmed that each immune cell type differentially responded during “early” [14,15] or “late” (24 h after injection) time periods. Interestingly, lymphocytes and white blood cells showed a genotype effect, suggesting that low vitamin E status may alter circulating immune cell profiles at 24 h post-injection. It is possible that there were different inflammatory responses between *Ttpa*^*–/–*^ and WT mice because *Ccl2* expression tended to be higher in control-*Ttpa*^*–/–*^ mice than WT mice (3.2 ± 2.1 compared with 1.0 ± 0.1; n.s.). Moreover, different plasma vitamin E levels between genotypes could cause an altered immune cell profile because immune cells contain many fold higher αT concentrations than red blood cells *in vitro* [42] suggesting that αT content in immune cells is important for optimal immune. αT plays a role in lymphocyte proliferation, helper T cell activity, and IL-2 production in mice [[43], [44], [45]]. Finno et al. [6,46] showed in *Ttpa*^*–/–*^ mice with poor αT status display elevated oxidative stress and altered expression of innate immune response markers. To obtain a more detailed understanding of the inflammatory response induced by stressors during αT depletion, future studies should employ flow cytometry to probe immune cell production or immune cell analysis performed on cultured leukocytes or from isolated macrophages following peritoneal lavage. Additionally, histological analysis of the brain regions to assess tissue damage and inflammatory cell infiltration would be beneficial to probe the impact of αT deficiency with and without LPS treatment on these outcomes.

We did not find any difference in inflammatory responses between WT and *Ttpa*^*–/–*^ mice. There could be several reasons for this: *1*) LPS triggers not only an oxidative stress response but also it could affect many signaling and gene expression pathways via Toll-like receptor (TLR). LPS stimulates TLR4 by enhancing the innate immune system which is induced by pathogen-associated molecular patterns [47], then inducing the production of proinflammatory cytokines. Although vitamin E supplementation enhanced recovery from LPS-induced sickness behavior and cytokine production in mice [22], αT itself does not directly activate the TLR4 pathway [48]. *2*) Synergistic effects with other nutrients, such as vitamin C or selenium, may impact the results. Gpx4 is vital for the prevention of lipid peroxidation in the liver [49]. Vitamin E can compensate for Gpx4 loss in murine liver and endothelium in vivo [49,50]. However, in this study, we did not see a difference in *Gpx4* expression either by LPS or vitamin E status. This result could be that another nutrient, such as selenium, may affect the enzyme expression and activity [51]. *3*) α-TTP is mainly expressed in the liver but also found in extrahepatic tissues, such as the brain, lung, kidney, spleen, and placenta [4,8,[52], [53], [54]]. The specific localization of α-TTP may regulate αT turnover, by protecting αT from degradation and effectively transporting αT to specific cell types, such as astrocytes in the brain. Although the mechanism is still uncertain, the brain may have its protection against any infection or oxidative stress. *4*) Due to the small sample size, the power of the sample size to determine the main effect of genotype was low (*β* = ∼0.75) for most of the endpoints.

Further studies will be needed to investigate the development and progression of chronic inflammation and oxidative stress following a single intraperitoneal LPS injection in mice. Twenty-four hours after injection may not be the most appropriate time to compare with the acute inflammatory phase. Time points could range from days to weeks to assess LPS-induced chronic inflammatory response because 24 h after LPS injection might be considered as part of the “acute” phase [37]. Multiple injections might be also needed to observe “chronic” responses in LPS-treated mice [55].

In summary, in this study, we evaluated several biochemical and functional endpoints 4 h (acute) or 24 h (partial recovery) after LPS injection following a 12-wk vitamin E depletion period. It was observed that partial recovery responses in both *Ttpa*^*–/–*^ and WT mice differed depending on the time tested following dosing. Grip strength was depressed at 4 h but not after 24 h for both genotypes. In contrast, after 24 h, but not 4 h after injection, we observed increased markers of an indirect oxidative stress response and altered immune cell counts in both genotypes. Despite not observing genotype effects at either timepoint, these findings inform additional studies regarding the timing of sampling for different endpoints.

## Author contributions

The authors’ responsibilities were as follows – MHS, AJS, JWE: designed research; MHS: conducted research; JWE: provided essential materials; MHS: analyzed data; MHS, JWE: wrote the article; MHS, AJS, JWE: contributed to manuscript revisions; JWE: had primary responsibility for final content; and all authors: read and approved the final manuscript.

## Conflict of interest

The authors report no conflicts of interest.

## Funding

This research was supported by Abbott Nutrition through the Center for Nutrition, Learning, and Memory at the University of Illinois, a USDA Hatch grant, the Division of Nutritional Sciences Vision 20/20, and the Margin of Excellence Research Program at the University of Illinois.

## Data availability

Data described in the manuscript will be made available upon request once the manuscript is published.
