# Supplementary material for: The Impact of LPS on Inflammatory Responses in Alpha-Tocopherol Deficient Mice
Source: Curr Dev Nutr. 2024 Jul 14;8(8):104416. doi: 10.1016/j.cdnut.2024.104416 (PMC11342875; doi:10.1016/j.cdnut.2024.104416)
Supplement: Multimedia component 1 [file mmc1.docx]

**SUPPLEMENTAL MATERIAL**

**Supplemental Table 1** Modified AIN-93G basal diet composition and content of oil-extracted α-tocopherol acetate added to experimental diets.^1^

| **Ingredient (g/kg diet)** | **αT deficient diet** | **LOW** |
| --- | --- | --- |
| Casein | 200 | 200 |
| L-Cystine | 3 | 3 |
| Corn starch | 398 | 398 |
| Maltodextrin | 132 | 132 |
| Sucrose | 100 | 100 |
| Cellulose | 50 | 50 |
| Soybean oil^2^ | 70 | 70 |
| t-butylhydroquinone | 0.01 | 0.01 |
| Mineral mix^3^ | 35 | 35 |
| Vitamin mix^4^ | 10 | 10 |
| Choline bitartrate | 2.5 | 2.5 |
| Novatol oil (86 % *RRR*-αTA)^5^ (mg/kg) | 0 | 0.035 |
| **Total αTA (mg/kg diet)** | ND^6^ | 36.6 |

^1^Diets were prepared by Research Diets (New Brunswick, NJ). The study mice (WT and *Ttpa^-/-^*) were fed an αT deficient diet for 12 weeks after weaning. *Ttpa* breeders were fed the LOW diet to require breeder reproduction but also minimize αT transfer to offspring.

^2^αT in soybean oil was 2.92 mg/kg diet via HPLC (Study mice should have received daily 5.8-8.8 ng αT derived from soybean oil based on average food intake, 2-3 grams/day).

^3^AIN-93 mineral mix for growing rodents (S10022G).

^4^AIN-93 vitamin mix with no vitamin E (V13402).

^5^The purity of αTA in the oil was analyzed via HPLC before diet production. The original purity from the company was 92.7 %, while the purity via HPLC was 80.3 %. We combined both purities to calculate the average αTA concentration.

^6^αTA levels were below the limit of detection (0.49 mg/kg) in the αT deficient diet.

Abbreviations: αT, α-tocopherol; αTA, α-tocopherol acetate; LOW, low vitamin E diet.


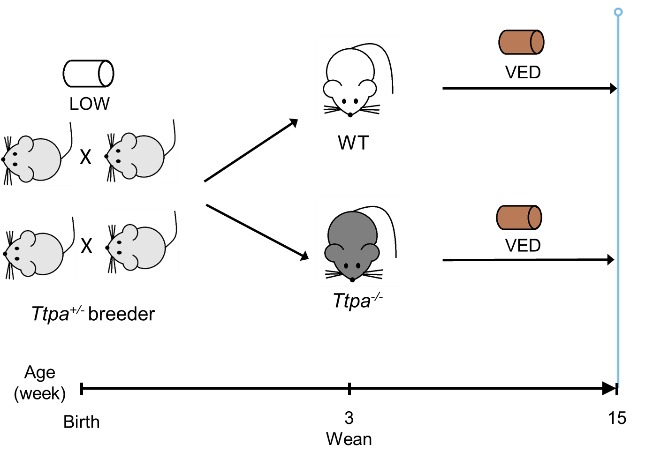
**Supplemental Figure 1. Breeding and study design protocol.**

Male *Ttpa^-/-^* and control WT littermates were generated using a trio-breeder strategy. The breeders were fed LOW diet. At weaning, the study mice were fed VED diet for 12 weeks. Abbreviations: LOW, low α-tocopherol; Ttpa, α-tocopherol transfer protein; VED, α-tocopherol deficient; WT, wild-type

**Supplemental Table 2** Primer sequences for RT-qPCR analysis in hippocampus and heart of WT and *Ttpa^-/-^* mice.^1^

| **Gene symbol** | **Forward** | **Reverse** |
| --- | --- | --- |
| *Actb* | GGCTGTATTCCCCTCCATCG | CCAGTTGGTAACAATGCCATGT |
| *aTub* | CAGGGCTTCTTGGTTTTCC | GGTGGTGTGGGTGGTGAG |
| *Ccl2* | CAGCAGGTGTCCCAAAGAAG | TGTGGAAAAGGTAGTGGATGC |
| *Cyp3a11* | CTCTCACTGGAAACCTGGGT | TCTGTGACAGCAAGGAGAGG |
| *Gpx1* | AGTCCACCGTGTATGCCTTCT | GAGACGCGACATTCTCAATGA |
| *Gpx4* | CACCCACTGTGGAAATGGAT | TGGTAAAGTTCCATTTGATGGC |
| *Gsr* | GTTTACCGCTCCACACATCC | TCCAGCTGAAAGAAGCCATC |
| *Il-6* | CTTCCATCCAGTTGCCTTCTTG | AATTAAGCCTCCGACTTGTGAAG |
| *Sod1* | CAGCATGGGTTCCACGTCCA | CACATTGGCCACACCGTCCT |
| *Tnf* | CTTCTGTCTACTGAACTTCGGG | CAGGCTTGTCACTCGAATTTTG |

^1^Primer sequences (5′ to 3′) were selected using previous publications, PrimerBank, and the IDT RealTime qPCR Assay tool.

**Supplemental Figure 2. Effect of vitamin E deficiency on body mass**


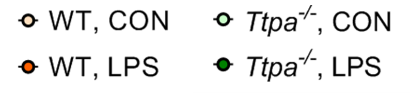

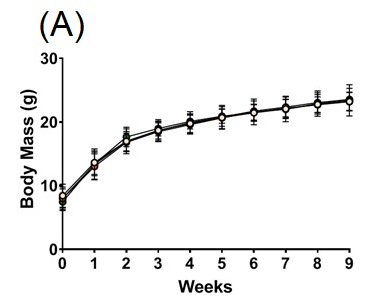

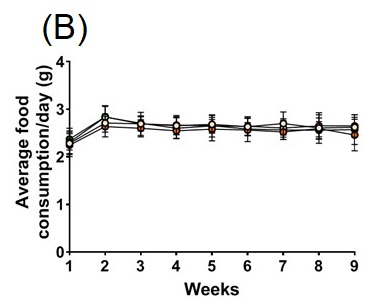


Weekly body mass (A) and average food intake/day (B) of WT and *Ttpa^-/-^* mice over the 12-week study period in **Study 1** (4 hr post-injection). Values are expressed as mean ± SEM. There were no significant differences between genotypes or treatment groups for body mass or average food intake, assessed by 3-way repeated measures ANOVA (n = 27 - 37/genotype). Additionally, there was no significant interaction (Study week x Genotype, Study week x LPS, Genotype x LPS, or Study week x Genotype x LPS) in body mass and food intake throughout the study periods. Abbreviations: CON, control; LPS, lipopolysaccharide; Ttpa, α-tocopherol transfer protein; WT, wild-type

**Supplemental Figure 3. Effects of vitamin E deficiency on the acute phase response to LPS injection.**


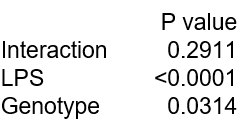

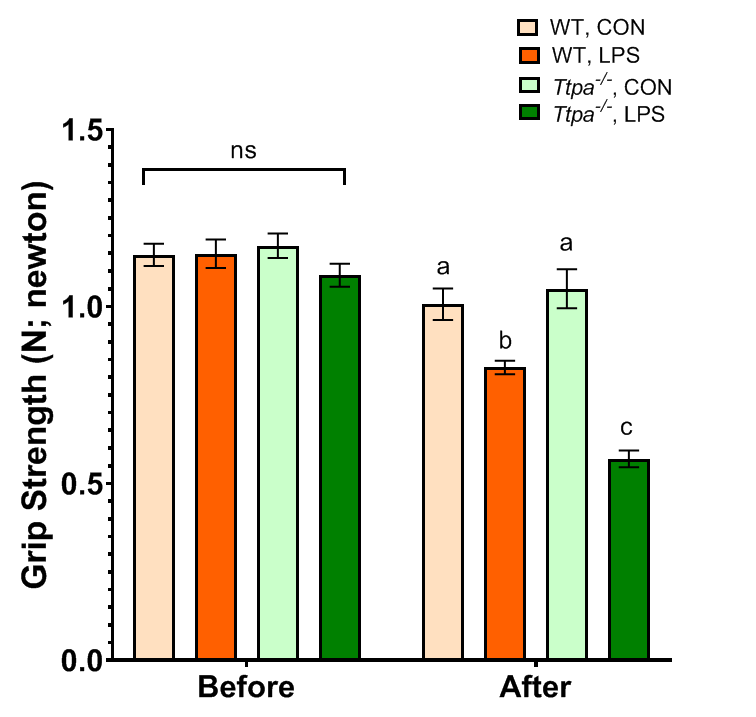

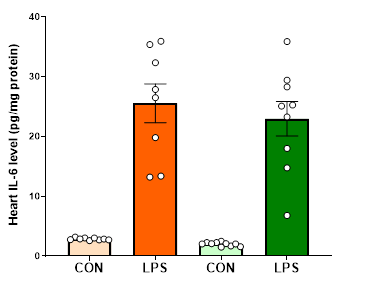


Male WT and *Ttpa^-/-^* mice were fed a αT deficient diet for twelve weeks, then inoculated with saline or LPS (10µg/mouse) and sacrificed 4 hours later. All capital IL-6 is represented by protein expression. Values, mean ± SEM pg/mg protein, of IL-6 levels in heart in male *Ttpa^-/-^* and WT mice (n=8-10/group) are shown. A 2 x 2 factorial ANOVA was conducted. Lower limit of
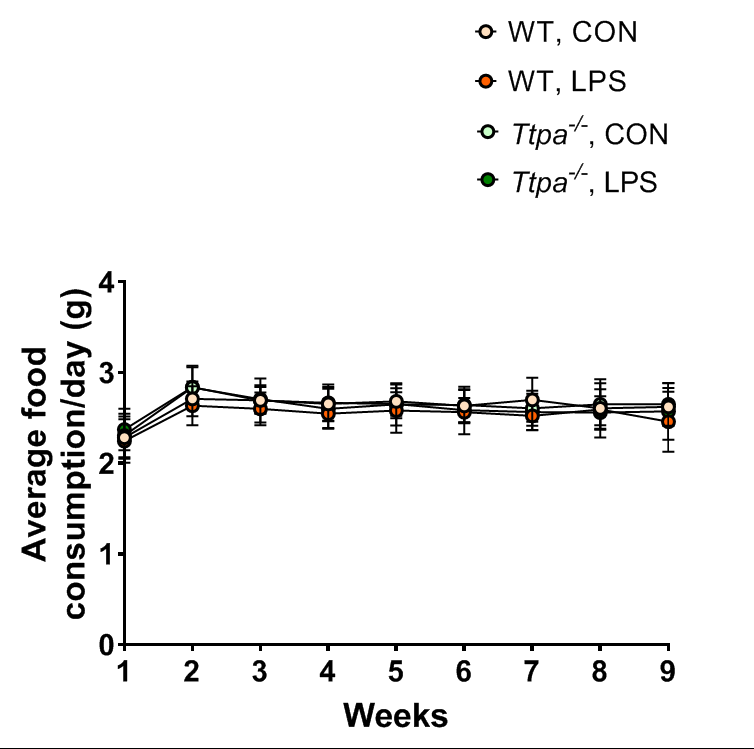
detection: 2 pg/ml. Abbreviations: CON, control; LPS, lipopolysaccharide; *Ttpa*, α-tocopherol transfer protein; WT, wild-type.

**Supplemental Table 3.** The proportion of circulating immune cells after 4-hour LPS injection between treatment groups.^1^

|  | **4-hour post-injection** | | | |
| --- | --- | --- | --- | --- |
|  | **WT** | | ***Ttpa^-/-^*** | |
| **Cell type (%)** | **CON** | **LPS** | **CON** | **LPS** |
| Neutrophils | 21.1 ± 1.2 | 39.5 ± 1.0 | 19.2 ± 1.1 | 37.5 ± 1.3 |
| Lymphocytes | 70.9 ± 1.7 | 49.0 ± 1.4 | 73.9 ± 1.4 | 49.0 ± 2.1 |
| Monocytes | 4.6 ± 0.5 | 3.2 ± 0.3 | 4.0 ± 0.4 | 4.8 ± 0.7 |
| Eosinophils | 2.6 ± 0.4 | 6.4 ± 0.5 | 2.2 ± 0.2 | 6.9 ± 0.8 |
| Basophils | 0.8 ± 0.1 | 2.0 ± 0.3 | 0.7 ± 0.1 | 1.8 ± 0.3 |

^1^Values are expressed as mean *±* SEM (%, n = 13-19/group).

Abbreviations: CON, control; LPS, lipopolysaccharide; Ttpa, α-tocopherol transfer protein; WT, wild-type.

**Supplemental Figure 4. Effect of vitamin E deficiency on body mass.**


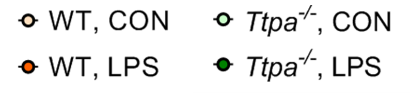

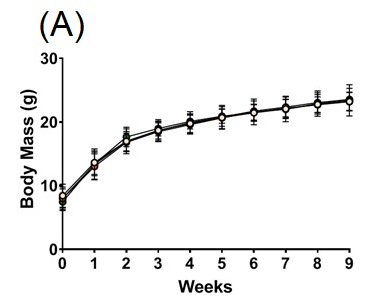

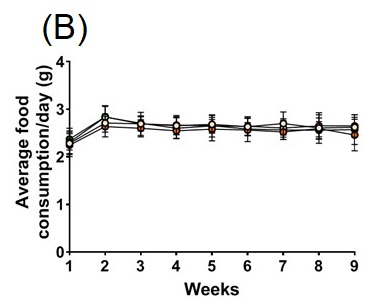


Weekly body mass (A) and average food intake/day (B) of WT and *Ttpa^-/-^* mice over the 12-week study period (**Study 2:** 24 hr post-injection). Values are expressed as mean ± SEM. There were no significant differences between genotypes or treatment groups for body mass or average food intake, assessed by repeated measures 3-way ANOVA (n = 23-31/genotype). Additionally, there was no significant interaction (Study week x Genotype, Study week x LPS, Genotype x LPS, or Study week x Genotype x LPS) in body mass and food intake throughout the study periods. Abbreviations: CON, control; LPS, lipopolysaccharide; Ttpa, α-tocopherol transfer protein; WT, wild-type

**Supplemental Table 4.** The proportion of circulating immune cells after 24-hour LPS injection between treatment groups.^1^

|  | **24-hour post-injection** | | | |
| --- | --- | --- | --- | --- |
|  | **WT** | | ***Ttpa^-/-^*** | |
| **Cell type** | **CON** | **LPS** | **CON** | **LPS** |
| Neutrophils | 12.7 ± 0.6 | 62.3 ± 4.3 | 16.0 ± 1.3 | 62.6 ± 3.9 |
| Lymphocytes | 80.2 ± 1.9 | 27.0 ± 4.3 | 80.2 ± 1.5 | 26.6 ± 3.9 |
| Monocytes | 2.2 ± 0.2 | 4.3 ± 0.4 | 2.1 ± 0.3 | 5.0 ± 0.3 |
| Eosinophils | 3.7 ± 0.9 | 3.1 ± 0.3 | 1.3 ± 0.2 | 3.8 ± 0.5 |
| Basophils | 1.3 ± 0.3 | 3.3 ± 1.1 | 0.4 ± 0.1 | 2.1 ± 0.2 |

^1^Values are expressed as mean *±* SEM (%, n = 10-13/group).

Abbreviations: CON, control; LPS, lipopolysaccharide; Ttpa, α-tocopherol transfer protein; WT, wild-type.
